# Supplementary material for: CRISPR artificial splicing factors
Source: Nat Commun. 2020 Jun 12;11:2973. doi: 10.1038/s41467-020-16806-4 (PMC7293279; doi:10.1038/s41467-020-16806-4)
Supplement: Supplementary file 1 — Supplementary Information [file 41467_2020_16806_MOESM1_ESM.pdf]

CRISPR Artificial Splicing Factors

Supplementary Information

Du and Jillette *et al*.

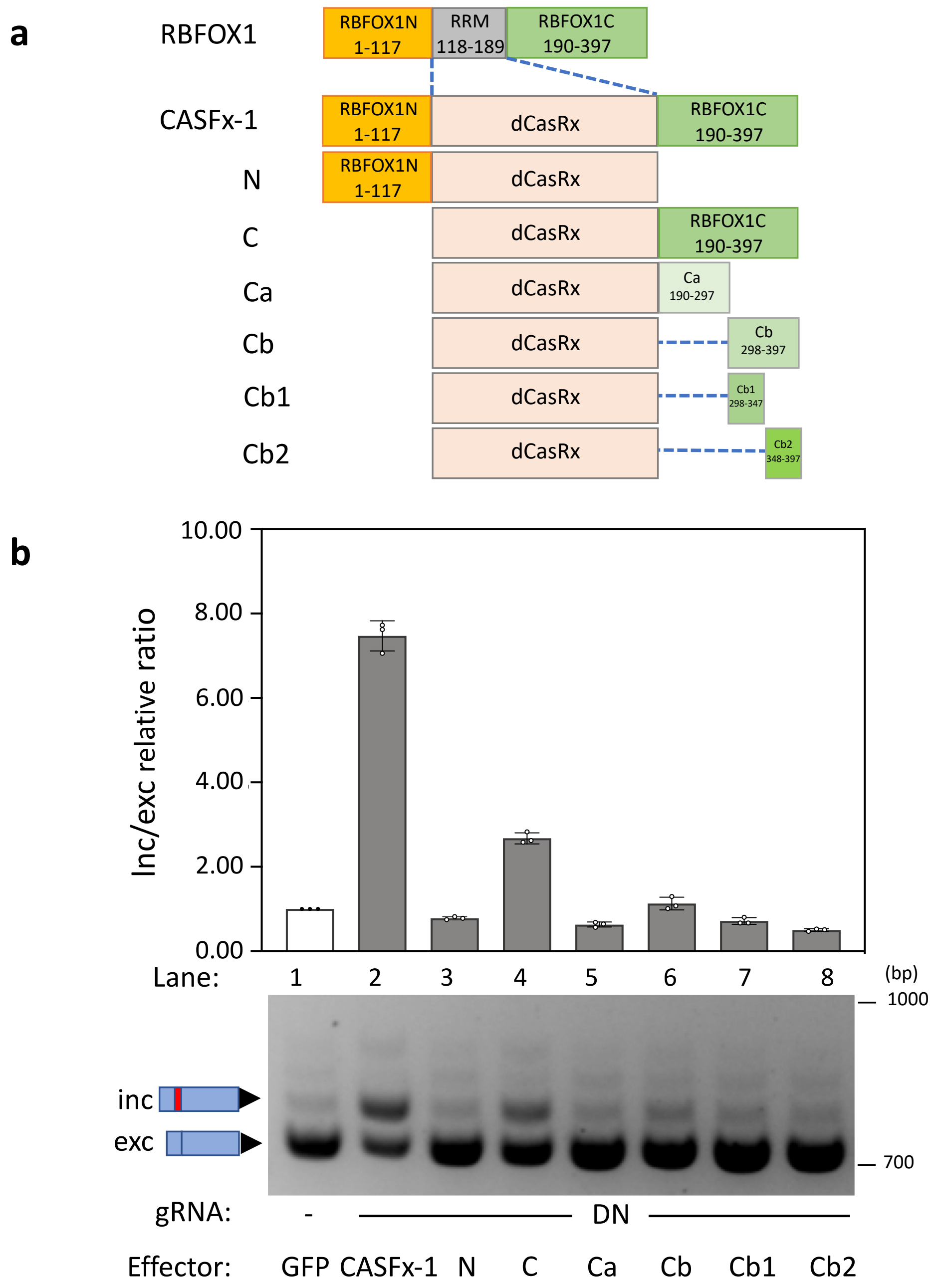

Supplementary Fig 1. Examining functional domains of RBFOX1 required for CASFx-1 splicing activity. (a) Diagrams of the RBFOX1, full length CASFx-1 (RBFOX1N-dCasRx-C) and RBFOX1 truncation mutants. (b) Upper panel shows inclusion/exclusion relative ratio fold-change assayed by qRT-PCR on *SMN2* minigene transcripts in cells co-transfected with control GFP plasmid (pmaxGFP), CASFx-1 (RBFOX1N-dCasRx-C) or

RBFOX1 truncation mutants, and the DN gRNAs, relative to that of the GFP control (set to 1). Data are represented as mean ± SD (n = 3). Lower panel shows a gel image of semi-quantitative splicing RT-PCR in the corresponding samples. The image shown is representative of two independent experiments. Uncropped gel images and qRT-PCR values are included in Source Data file.

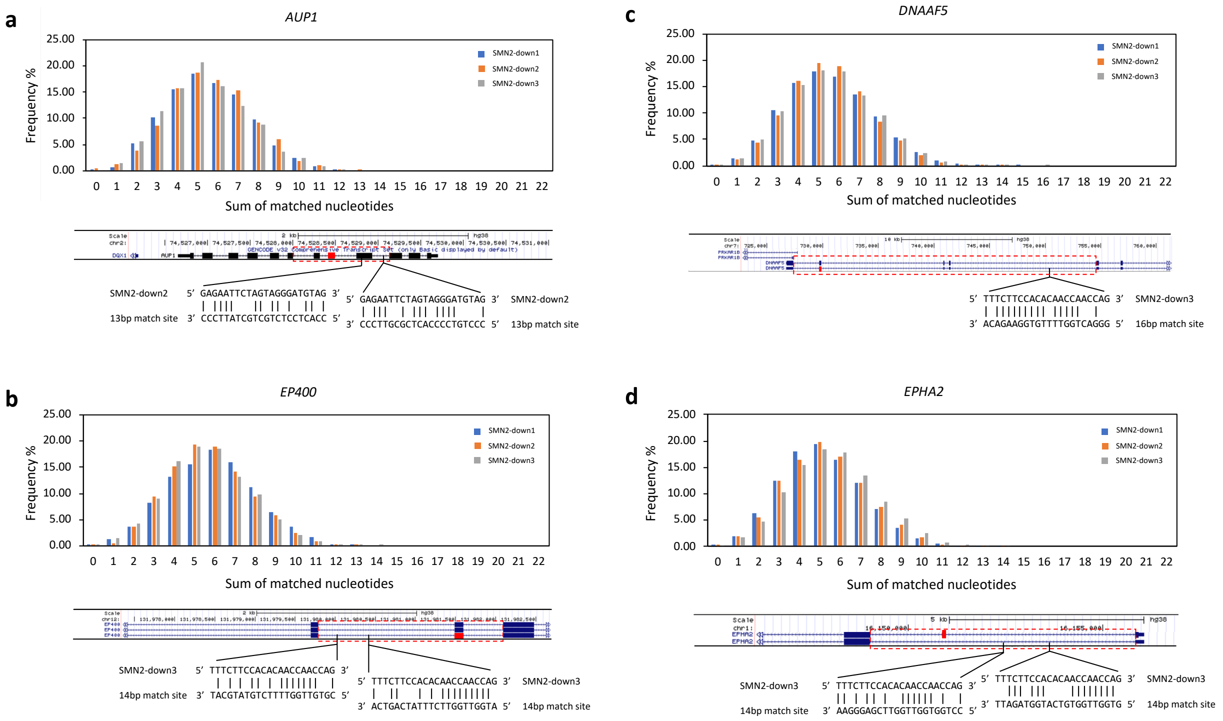

Supplementary Fig 2. Sequence match profiles of the off-target events induced by CASFx-1. To calculate the sum of matched nucleotides, three gRNA spacers were compared against a search window encompassing the upstream intron, cassette exon, and downstream intron of the off-target events (highlighted by the dotted rectangle). (a) Upper panel shows number of positions (y-axis) with the indicated number of matching nucleotides (x-axis) within the search window on *AUP1* for each *SMN2* gRNA. The bottom panel shows the two sites sharing 13 bp matched sequence between SMN2-down2 and the intron region. The cassette exon is marked in red. (b) Upper panel shows number of positions (y-axis) with the indicated number of matching nucleotides (x-axis) within the search window on *EP400* for each *SMN2* gRNA. The bottom panel shows the two sites sharing 14 bp matched sequence between SMN2-down3 and the intron region. The cassette exon is marked in red. (c) Upper panel shows number of positions (y-axis) with the indicated number of matching nucleotides (x-axis) within the

search window on *DNAAF5* for each *SMN2* gRNA. The bottom panel shows the site sharing a 16 bp matched sequence between SMN2-down3 and the intron region. The cassette exon is marked in red. (d) Upper panel shows number of positions (y-axis) with the indicated number of matching nucleotides (x-axis) within the search window on *EPHA2* for each *SMN2* gRNA. The bottom panel shows the two sites sharing 14 bp matched sequence between SMN2-down3 and the intron region. The cassette exon is marked in red.

a

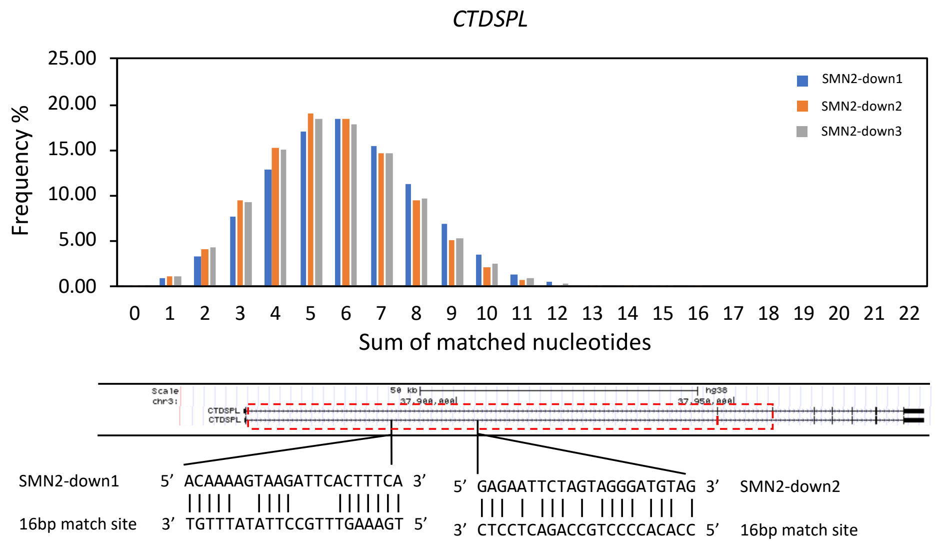

b

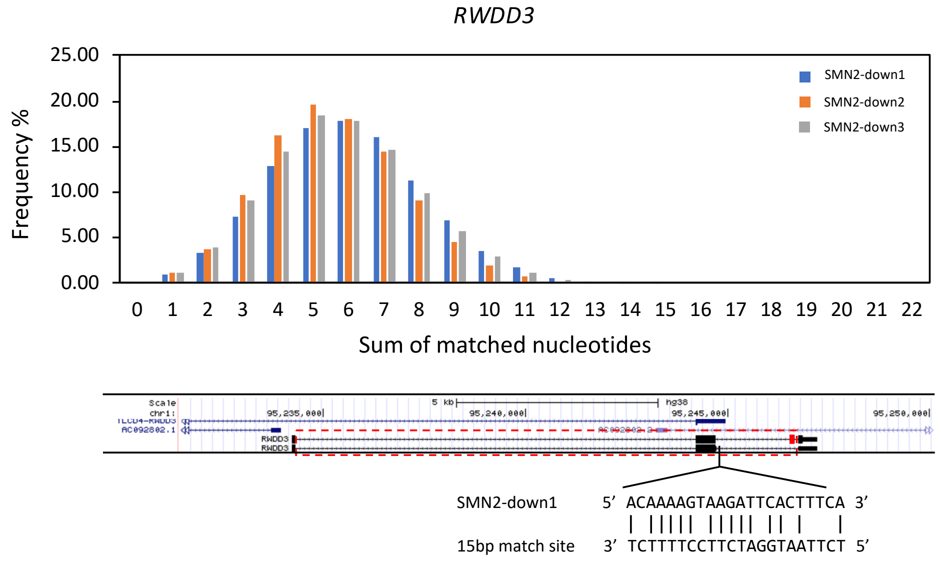

Supplementary Fig 3. Sequence match profiles of the off-target events induced by CASFx-3. To calculate the sum of matched nucleotides, three gRNA spacers were compared against a search window encompassing the upstream intron, cassette exon, and downstream intron of the off-target events (highlighted by the dotted rectangle). (a) Upper panel shows number of positions (y-axis) with the indicated number of matching nucleotides (x-axis) within the search window on *CTDSPL* for each *SMN2* gRNA. The

bottom panel shows the two sites sharing 16 bp matched sequence between SMN2-down1 or SMN2-down2 and the intron region. The cassette exon is marked in red. (b) Upper panel shows number of positions (y-axis) with the indicated number of matching nucleotides (x-axis) within the search window on *RWDD3* for each *SMN2* gRNA. The bottom panel shows the site sharing 15 bp matched sequence between SMN2-down1 and the intron region. The cassette exon is marked in red.

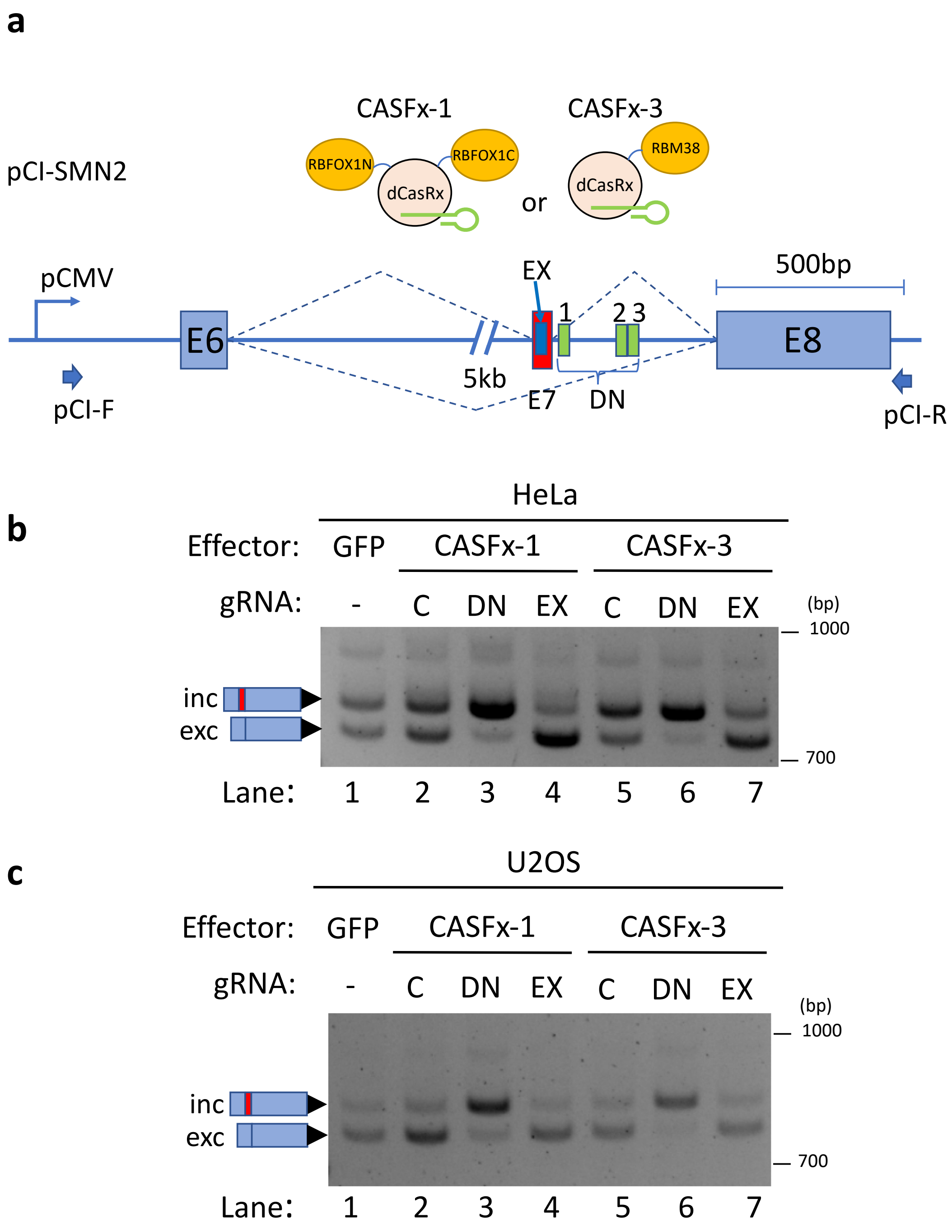

Supplementary Fig 4. Activation and repression of SMN2-E7 by differential positioning of CASFx in HeLa and U2OS cells. (a) Schematic of the CRISPR artificial splicing factors, CASFx-1 (RBFOX1N-dCasRx-C), CASFx-3 (dCasRx-RBM38) and *SMN2* minigene, as well as a set of three target sites downstream of E7 (DN: gRNA-1 through

3) and one target site target within E7 (EX). (b) Gel image of semi-quantitative splicing RT-PCR of the corresponding transfected samples in HeLa cells. “C” indicates a control gRNA without matching *SMN2* minigene sequence. The image shown is representative of two independent experiments. (c) Gel image of semi-quantitative splicing RT-PCR of the corresponding transfected samples in U2OS cells. “C” indicates a control gRNA without matching *SMN2* minigene sequence. The image shown is representative of two independent experiments. Uncropped gel images are included in Source Data file.

a

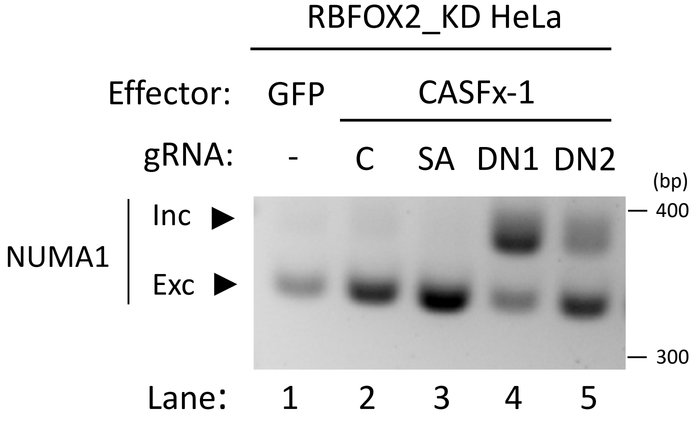

b

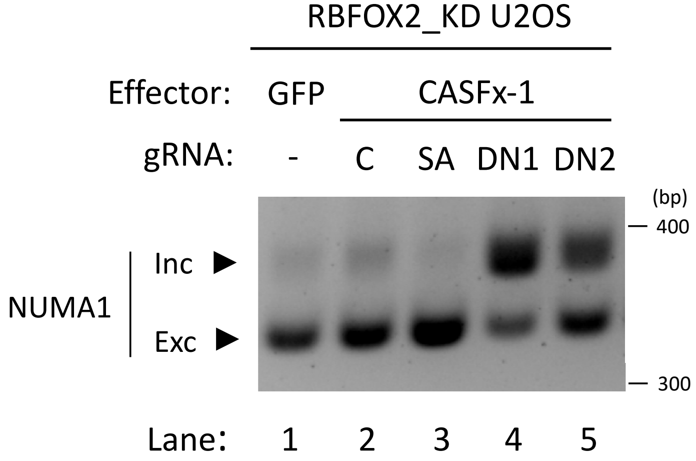

Supplementary Fig 5. Using CASFx-1 to activate cassatte exon of *NUMA1*, a target of RBFOX1/2, in RBFOX2 knockdown (RBFOX2_KD) HeLa and U2OS cells. RBFOX2_KD cells were transfected with *NUMA1* minigene, CASFx-1 (RBFOX1N-dCasRx-C) and indicated gRNAs. (a) Gel image of semi-quantitative splicing RT-PCR of the corresponding transfected samples in RBFOX2_KD HeLa cells. “C” indicates a control gRNA without matching *NUMA1* minigene sequence. “SA” indicates a gRNA

targeting onto *NUMA1* exon. “DN1” and “DN2” represent two gRNAs targeting to downstream sequence of *NUMA1* exon. The image shown is representative of two independent experiments. (b) Gel image of semi-quantitative splicing RT-PCR of the corresponding transfected samples in RBFOX2_KD U2OS cells. The image shown is representative of two independent experiments. Uncropped gel images are included in Source Data file.

Supplementary Table 1. Sequences of gRNAs used in this study

Target site	gRNA name	gRNA sequence (5'-3')
Non-target control	Control	AGCAGCAGCAGCAGCAGCAGCA
SMN2 minigene downstream of exon 7	SMN2-down1	ACAAAAGTAAGATTCACTTTCA
	SMN2-down2	GAGAATTCTAGTAGGGATGTAG
	SMN2-down3	TTTCTTCCACACAACCAACCAG
	SMN2-down4	GCTGGTCTGCCTACTAGTGATA
SMN2 minigene exon 7	SMN2-EX	AATGTGAGCACCTTCCTTCTTT
RG6 minigene exon CX	RG6-SA	ATATCGCCTGGATCCTGAGCCA
SMN2 minigene downstream of exon 7	SMN2-DN	AACCCCTACCAACTGGTCGGGGTTTGAAACACAAAAGTAAGATTCACTTTCACAAGTAAACCCCTACCAACTGGTCGGGGTTTGAAACGAGAATTCTAGTAGGGATGTAGCAAGTAAACCCCTACCAACTGGTCGGGGTTTGAAACTTTCTTCCACACAACCAACCAG (DR, gRNA)
SMN2 minigene downstream of exon 7 and *RG6* minigene exon CX	SMN2-DN-RG6-SA	AACCCCTACCAACTGGTCGGGGTTTGAAACACAAAAGTAAGATTCACTTTCACAAGTAAACCCCTACCAACTGGTCGGGGTTTGAAACGAGAATTCTAGTAGGGATGTAGCAAGTAAACCCCTACCAACTGGTCGGGGTTTGAAACTTTCTTCCACACAACCAACCAGCAAGTAAACCCCTACCAACTGGTCGGGGTTTGAAACATATCGCCTGGATCCTGAGCCA (DR, gRNA)
Endogenous *SMN2* downstream of exon 7<br><br>(all-in-one RBFOX1N-dCasRx-C)	SMN2-down1_2_3	AACCCCTACCAACTGGTCGGGGTTTGAAACACAAAAGTAAGATTCACTTTCACAAGTAAACCCCTACCAACTGGTCGGGGTTTGAAACGAGAATTCTAGTAGGGATGTAGCAAGTAAACCCCTACCAACTGGTCGGGGTTTGAAACTTTCTTCCACACAACCAACCAG (DR, gRNA)

Endogenous *SMN2* downstream of exon 7<br><br>(all-in-one RBFOX1N-dPspCas13b-C)	SMN2-down4_5_6	GTAAGATTCACTTTCATAATGCGTTGTGGAAGGTCCAGTTTTGAGGGGCTATTACAACGTAGGGATGTAGATTAACCTTTGTTGTGGAAGGTCCAGTTTTGAGGGGCTATTACAACGCTGGTCTGCCTACTAGTGATAGTTGTGGAAGGTCCAGTTTTGAGGGGCTATTACAAC (DR, gRNA)
NUMA1 minigene	NUMA1-down1	CAGCCAGCATTTAGCGAGGACC
	NUMA1-down2	GCCGGGCTCATGCACCAAGACT
	NUMA1-SA	AGCTGCTCCACCTGTACATGGG

Supplementary Table 2. List of PCR primers used in this study

Target site	Primer name	Primer sequence (5'-3')	Experiment
SMN2 minigene	pCI-F	GCTAACGCAGTCAGTGCTTC	RT-PCR
	pCI-R	GTATCTTATCATGTCTGCTCG	
RG6 minigene	RG6-F	ATGGATTACAAGGATGACGATGAC	RT-PCR
	RG6-R	GCGCATGAACTCCTTGATGAC	
Endogenous *SMN2*	En-F	ACCACCTCCCATATGTCCAG	RT-PCR
	En-R	TTTTGAAGAAATGAGGCCAGTT	
SMN2 minigene	Vector-F	GCTCTTAAGGCTAGAGTACTTAATACGA	Quantitative RT-PCR
	Exclusion-R	CTTCTTTTTGATTTTGTCTAAAACCCATATAATAG	
	Inclusion-R	CTCTATGCCAGCATTTCCATATAATAG	
RG6 minigene	Exclusion-F	CGGATCTAGAGGTCAGGAGGA	Quantitative RT-PCR
	Inclusion-F	GAAGAATTCAGGTCAGGAGGA	
	Vector-R	GGTGTTGTGGCCCTCGTAG	
Endogenous *SMN2*	Endo-F	ATAATTCCCCCACCACCTC	Quantitative RT-PCR
	Exclusion-R	CTTCTTTTTGATTTTGTCTAAAACCCATATAATAG	
	Inclusion-R	CTCTATGCCAGCATTTcCATATAATAG	

Supplementary Table 3. Lists of off-target exon skipping (SE) events identified from RNA-seq of CASFx-1, CASFx-3 and ESF mix samples.

CASFx-1								
Gene Symbol	chr	strand	Exon Start	Exon End	Upstream ExonEnd	Downstream ExonStart	FDR	InclusionLevel Difference
AUP1	chr2	-	74528416	74528489	74528006	74529131	9.57E-05	-0.583
EP400	chr12	+	131981488	131981596	131979793	131982092	8.71E-04	-0.271
DNAAF5	chr7	+	729662	729847	727315	754588	1.08E-03	-0.119
EPHA2	chr1	-	16150895	16150963	16149047	16155847	5.41E-03	-0.185

CASFx-3								
Gene Symbol	chr	strand	Exon Start	Exon End	Upstream ExonEnd	Downstream ExonStart	FDR	InclusionLevel Difference
CTDSPL	chr3	+	37947056	37947211	37862278	37964570	8.91E-03	-0.197
RWDD3	chr1	+	95246541	95246657	95234315	95246755	8.16E-03	0.186

ESF mix								
Gene Symbol	chr	strand	Exon Start	Exon End	Upstream ExonEnd	Downstream ExonStart	FDR	InclusionLevel Difference
TPM3	chr1	-	154171412	154171488	154170711	154172907	1.17E-07	0.728
VDAC2	chr10	+	75217887	75217991	75212298	75219062	1.47E-05	0.55
GUSB	chr7	-	65979398	65979541	65967907	65981973	5.65E-04	0.458
PPP2R5B	chr11	+	64925470	64925933	64925028	64927801	6.84E-07	0.403
TMEM51	chr1	+	15210489	15210562	15153954	15214894	1.03E-03	-0.361
CCT6P1	chr7	+	65759174	65759271	65758317	65759859	3.95E-07	0.303
ABI1	chr10	-	26759061	26759235	26751783	26765217	1.72E-07	0.283
ABI1	chr10	-	26755654	26755741	26751783	26765217	2.91E-06	0.278
SNHG12	chr1	-	28579911	28579981	28579587	28581589	4.04E-07	0.274

DDB2	chr11	+	47232813	47232959	47217049	47237836	5.55E-05	0.269
GADD45A	chr1	+	67686024	67686126	67685538	67687660	1.00E-08	0.268
TIMM23B	chr10	+	49945031	49945090	49942300	49952125	5.45E-03	0.268
GUSB	chr7	-	65976014	65976202	65967907	65981973	1.93E-04	0.26
NRM	chr6	-	30689275	30689452	30688942	30690841	5.36E-07	0.251
ARHGEF11	chr1	-	156938417	156938513	156937496	156939547	3.47E-04	0.246
CROCCP2	chr1	-	16626352	16626499	16626005	16627144	7.61E-04	0.24
SNHG12	chr1	-	28580559	28580646	28579981	28581589	4.55E-05	0.231
NSUN5P1	chr7	+	75412788	75412932	75410460	75414884	2.11E-03	-0.221
ABI1	chr10	-	26759061	26759238	26751783	26765217	3.05E-07	0.22
DDB2	chr11	+	47234572	47234672	47232959	47235269	3.05E-07	0.205
CTTN	chr11	+	70421469	70421580	70420510	70422939	1.00E-08	0.201
NSUN5P2	chr7	-	72952151	72952295	72950196	72954624	2.29E-04	-0.201
FANCG	chr9	-	35074370	35074494	35074216	35075278	1.00E-08	0.17
FANCG	chr9	-	35074926	35075082	35074216	35075278	1.00E-08	0.169
TMEM180	chr10	+	102470664	102471117	102470075	102471272	9.11E-06	0.165
TSC22D4	chr7	-	100477276	100477462	100474440	100478793	7.42E-04	0.158
ILKAP	chr2	-	238183652	238183740	238182186	238184019	1.00E-08	0.156
PTS	chr11	+	112230207	112230230	112228673	112233162	2.03E-05	0.156
RAD54L	chr1	+	46259963	46260099	46258746	46261260	1.58E-08	0.155
TSC22D4	chr7	-	100477276	100477459	100474440	100478793	4.17E-04	0.155
SNHG12	chr1	-	28581110	28581229	28579981	28581589	9.81E-06	0.152
TBRG4	chr7	-	45104537	45104709	45103443	45105440	2.32E-08	0.15
ZNF316	chr7	+	6637845	6638009	6637447	6639041	2.02E-03	0.15
PI4KB	chr1	-	151326171	151326373	151307801	151327270	5.48E-03	0.149
NSUN5	chr7	-	73308430	73308553	73305097	73308697	1.00E-08	0.142
NRM	chr6	-	30690024	30690243	30688942	30690841	3.14E-04	0.142

PTS	chr11	+	112230625	112230682	112228673	112233162	9.81E-06	0.14
TBCE	chr1	+	235419416	235419561	235414618	235427139	2.34E-03	0.139
TBRG4	chr7	-	45104098	45104256	45103443	45105440	3.05E-07	0.137
VARS	chr6	-	31782284	31782443	31782177	31782720	1.00E-08	0.135
TSC22D4	chr7	-	100477276	100478204	100474440	100478793	1.43E-04	0.133
SLMAP	chr3	+	57925844	57925934	57923023	57927295	2.22E-04	0.133
PXDC1	chr6	-	3727550	3727662	3723736	3737078	2.10E-04	-0.129
ODF2	chr9	+	128460549	128460666	128459657	128460941	7.74E-04	0.128
FYN	chr6	-	111699514	111699670	111696456	111702884	9.31E-04	0.128
NRM	chr6	-	30690046	30690243	30688942	30690841	4.64E-04	0.127
TSC22D4	chr7	-	100477276	100478307	100474440	100478793	1.57E-04	0.122
RAD54L	chr1	+	46260726	46261015	46258746	46261260	2.52E-08	0.121
GADD45A	chr1	+	67686349	67686587	67685538	67687660	1.00E-08	0.115
RIT1	chr1	-	155910655	155910804	155910506	155911242	1.30E-03	0.115
EIF2D	chr1	-	206599462	206599612	206599092	206600262	1.25E-05	0.111
FGFR1OP	chr6	+	167003722	167003811	167003247	167004263	9.05E-03	0.111
ALAS1	chr3	+	52208082	52208247	52206751	52211282	1.02E-08	0.109
TRIM11	chr1	-	228400963	228401194	228397165	228402065	1.52E-04	0.105
EIF2D	chr1	-	206599732	206599836	206599092	206600262	9.81E-06	0.104
NOP16	chr5	-	176386839	176386909	176385327	176388432	5.67E-04	0.103
SARS	chr1	+	109230877	109231021	109229572	109231630	1.00E-08	0.102
RETSAT	chr2	-	85351679	85351862	85351021	85354335	3.72E-08	0.101
POLH	chr6	+	43582315	43582456	43576440	43583006	3.65E-03	0.101
